# LVAD therapy as a catalyst to heart failure remission and myocardial recovery

**DOI:** 10.1002/clc.24094

**Published:** 2023-08-01

**Authors:** Eman A. Hamad, Mirnela Byku, Sharon B. Larson, Filio Billia

**Affiliations:** ^1^ Lewis Katz School of Medicine Temple University Philadelphia Pennsylvania USA; ^2^ Department of Medicine University of North Carolina Chapel Hill North Carolina USA; ^3^ Baptist Heart Institute at Baptist Memorial Hospital Memphis Tennessee USA; ^4^ Peter Munk Cardiac Center University Health Network Toronto Ontario Canada

**Keywords:** explant, GDMT, LVAD, recovery

## Abstract

The management of chronic heart failure over the past decade has witnessed tremendous strides in medical optimization and device therapy including the use of left ventricular assist devices (LVAD). What we once thought of as irreversible damage to the myocardium is now demonstrating signs of reverse remodeling and recovery. Myocardial recovery on the structural, molecular, and hemodynamic level is necessary for sufficient recovery to withstand explant and achieve sustained recovery post‐LVAD. Guideline‐directed medical therapy and unloading have been shown to aid in recovery with the potential to successfully explant the LVAD. This review will summarize medical optimization, assessment for recovery, explant methodologies and outcomes post‐recovery with explant of durable LVAD.

## Graphical abstract

Myocardial recovery on the structural, molecular, and hemodynamic levels is necessary to withstand the explant for sustained recovery postleft ventricular assist device. Guideline‐directed medical therapy and unloading can aid in this process. This review will summarize evidence for reverse remodeling and myocardial recovery, and propose a protocol for optimization and assessment of myocardial recovery.

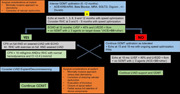

## 1 INTRODUCTION

While patients with heart failure (HF), who are optimized with guideline‐directed medical therapy (GDMT), do demonstrate an improvement in survival and hospitalizations, there remains 5% of patients who progress and develop advanced HF.^1^ In the REMATCH (Randomized Evaluation of Mechanical Assistance for the Treatment of Congestive Heart Failure) trial, left ventricular assist device (LVAD) therapy showed a great improvement in survival in stage D HF patients, compared to medical therapy.^2^ This coupled with the donor shortage has transformed the way we manage HF patients. LVAD therapy has become a great option for those who are not transplant candidates or are too sick to wait for a transplant without mechanical circulatory support. With improvement in LVAD technology and adverse event profile, clinical outcomes have improved, and longer and safer LVAD support duration can be offered. This was evident by the recent 5‐year outcomes of the landmark MOMENTUM 3 (Multicenter Study of MagLev Technology in Patients Undergoing Mechanical Circulatory Support Therapy with HeartMate 3) trial that evaluated the 5‐year outcomes and adverse event profile of the HeartMate 3 (HM3) LVAD, compared to its predecessor, the HeartMate II (HMII).^3^

The 5‐year Kaplan–Meier estimate of survival was 58.4% in the centrifugal‐flow group versus 43.7% in the axial‐flow group (hazard ratio: 0.72; 95% confidence interval [CI]: 0.58–0.89; *p* = .003). Serious adverse events of stroke, bleeding, and pump thrombosis were less frequent in the centrifugal‐flow pump group. As a result of longer support with less adverse events, we began to observe signs of myocardial recovery in some patients. This prompted further investigation into what constitutes myocardial recovery and factors contributing to “reversibility of myocardial dysfunction” in LVAD patients. Despite the rate of true recovery remaining low with a lack of evidence for its sustainability, developing a better understanding of what underlies myocardial recovery has become a major research focus in the field.

In the context of LVAD therapy, “true myocardial recovery” and “myocardial remission” are terms used to describe levels of cardiac function improvement in patients who receive mechanical support. True myocardial recovery refers to the restoration of native heart function to a degree where ongoing mechanical circulatory support is not needed. It indicates that the heart has regained its ability to adequately pump blood without assistance from the LVAD. In these cases, the LVAD can be explanted, and the patient can resume a normal life without further mechanical support. Myocardial remission, on the other hand, suggests an improvement in cardiac function but does not necessarily imply complete recovery to the extent where the LVAD can be removed. It indicates that the heart has shown some degree of recovery, potentially resulting in improved symptoms, ventricular function, and exercise capacity.^4^

Of note, the distinction between true myocardial recovery and myocardial remission can be challenging to determine since there is no agreed upon universal definition. This contributes to variations in the reported rates of recovery among different studies. Of note, the distinction between true myocardial recovery and myocardial remission can be challenging to determine since there is no agreed upon universal definition. This contributes to variations in the reported rates of recovery among different studies. While not all cases of HF can be reversed, some patients with reversible causes of cardiomyopathy such as tachycardia‐induced, peripartum, Takotsubo, alcohol, and chemotherapy‐induced cardiomyopathy may experience higher chances of sufficient improvement in cardiac function to allow for LVAD explantation.^5^

## 2 EVIDENCE FOR REVERSE REMODELING AND MYOCARDIAL REMISSION IN LVAD PATIENTS

### 2.1 Medical optimization for recovery and mechanical unloading

Based on the INTERMACS (Interagency Registry for Mechanically Assisted Circulatory Support) data, cardiac recovery during long‐term LVAD therapy is reportedly as low as 1.3%.^6^ Recovery of cardiac function post‐LVAD implant has been attributed to a reversal of the remodeling process, described as the regression or reversal of pathological hypertrophy, chamber shape distortion, and myocardial dysfunction.^7^ Addressing any reversible pathophysiologic processes that might impact reverse remodeling is important. These processes include ischemia, use of cardiotoxic agents, arrhythmias, thyroid disease, and alcohol usage. GDMT has been associated with reverse remodeling and improved patient outcomes.^8^ Left ventricular (LV) unloading is an additional key factor linked to recovery.^6^ As such, the combination of medical optimization and mechanical unloading has been shown to reduce HF readmissions.^9^, ^10^ Given the lack of guidelines for medical and mechanical optimization post‐LVAD, the number of patients with the potential for recovery is likely underestimated, thus presenting us with a missed opportunity. With the low rate of LV recovery and the lack of evidence for its sustainability, developing a better understanding of what underlies myocardial recovery has become a major research focus in the field.

Medical optimization with the goal of reverse LV remodeling should be considered in all patients supported with an LVAD. It is recommended that algorithms should be based on consensus documents for the use of GDMT, as per the 2021 update to the 2017 ACC Expert Consensus Decision Pathway for Optimization of Heart Failure Treatment.^11^ The optimization of GDMT post‐LVAD was correlated with reduced morbidity and mortality.^12^ Interestingly, higher sympathetic activity, as measured by norepinephrine levels, was found in LVAD patients not treated with angiotensin‐converting enzyme inhibitors postexplant for orthotopic heart transplan. This correlated with higher rates of fibrosis, myocardial stiffness, and LV mass, which suggests the absence of reverse remodeling in this group. Interestingly, LVAD patients treated with β‐blockers had lower N‐terminal pro b‐type natriuretic peptide (NT‐proBNP) levels and improved overall survival.^13^

There is limited data on angiotensin receptor‐neprilysin inhibitor (ARNI); however, the available data is promising. A single center analysis of 30 LVAD patients on ARNI showed a significantly lower NT‐proBNP level at 6 months and better control of blood pressure.^14^ The Harefield experience on the use of clenbuterol, a selective β2 agonist, has been associated with recovery and reverse remodeling when combined with mechanical unloading. However, these results have not been reproducible in any large‐scale clinical trial.^5^ The updated HF guidelines for medical optimization that includes sodium/glucose cotransporter‐2 inhibitor (SGLT2i) pose the question of benefit in the LVAD population. The safety of SGLT2i use among LVAD patients with type 2 diabetes mellitus has been discussed in a retrospective review.^15^ In addition, a clinical trial that is soon to be launched will be investigating SGLT2i in LVAD patients (https://clinicaltrials.gov/ct2/show/NCT05278962). It would be interesting to see if there is an added benefit to reverse remodeling and recovery.

Mechanical unloading of the failing heart is associated with changes at the structural, molecular, and cellular levels; all of which have been linked to functional improvement.^16^ The greatest degree of recovery in LV ejection fraction (LVEF) was reported at 6 months of unloading for patients with either ischemic or nonischemic cardiomyopathy, with a relative increase in EF to above 50%. This was reflected in an improvement in LV end‐systolic and end‐diastolic dimensions, diastolic parameters, and decreased LV mass, as early as 30 days.^16^ The improvement can persist to 1‐year.

LVAD unloading also leads to a decrease in circulating levels of many neurohormones (epinephrine, norepinephrine, arginine, vasopressin, renin, and angiotensin II).^17^, ^18^ This is consistent with the observations seen with GDMT. Other beneficial effects unloading has on the structure and function of the human heart are reflected in cellular and molecular changes and the activation of adrenergic and sympathetic pathways.^19^ Interestingly, there are also effects on electrical remodeling as reflected by the shortening of QRS and QT intervals post‐LVAD associated with an increase in LVEF and a decrease in filling pressures.^18^

Simon et al.^20^ evaluated the effects of LVAD therapy on reverse remodeling. Mechanical unloading with LVADs led to improvements in cardiac output, reduction in pulmonary artery pressure, and reverse remodeling of the left ventricle with improvement in structure and function. Additionally, neurohormonal imbalances associated with HF, such as elevated levels of natriuretic peptides and sympathetic activation, were improved after LVAD implantation.^20^ Moreover, mechanical unloading provided by LVADs has been shown to have a role in reducing wall stress and myocardial oxygen consumption. This emphasizes the importance of optimal medical therapy in conjunction with LVAD support to maximize reverse remodeling. Medical therapy aims to target neurohormonal imbalances and improve cardiac function, which, combined with mechanical unloading, can lead to improved cardiac output, blood pressure, and reverse remodeling.^21^

Finally, the process of reconditioning after unloading has also been observed. This entailed a slow decrease in speed once the aortic valve opening time reached 10% of the cardiac cycle at a low speed of 6000 for HMII representing signs of reverse remodeling.^22^ Reconditioning continued until valve opening time normalized. Taken together, the data on the combination of GDMT and unloading is indeed promising.

## 3 OUTCOMES IN CONTEMPORARY LVAD PATIENTS AND EVIDENCE FOR SUCCESSFUL EXPLANT

### 3.1 Sustainability of myocardial recovery and supportive evidence

The addition of standard GDMT to LVAD supports augments the suppression of neurohormonal activity, further promoting a reversal of LV structural changes; however, since the initial studies on reverse remodeling and myocardial unloading following LVAD support, there were concerns that complete normalization of both anatomic shape and function along with the reversal of the molecular changes are not achieved. This is coupled with the fact that only a few patients achieve resolution of the adverse neurohormonal status after LVAD explantation.^17^, ^23^, ^24^, ^25^ In addition, there are concerns about partial normalization in that the normalization of LV structure and function is accompanied by only a partial normalization of genetic and epigenetic changes.^26^, ^27^, ^28^, ^29^

After discontinuation of LVAD support, the risk of recurrent HF and other cardiovascular events has been shown to be highest in the first year. Long‐term survival free from LVAD, transplant, or death after LVAD explanation for myocardial recovery is reported ~90% at 1 year and 77% at 3 years postexplant.^30^ In patients with minimally invasive LVAD decommissioning, survival free from HF recurrence, LVAD reimplantation, or transplant at 1, 2, and 3 years was 94%, 87%, and 78%, respectively.^31^ In this study, while all patients were treated with β‐blockers and ACE/angiotensin II receptor blocker/ARNI, there were changes noted in LVEF and cardiac dimensions. Immediately after discontinuation of LVAD support, the LVEF decreased by 7%–40% and the LV internal diameter (LVIDd) increased by 0.4–5.2 cm. After 3 years, the LVEF and LVIDd were 42% and LVIDd of 5.8 cm, respectively. Another important consideration related to driveline infection.^31^ Before decommissioning, eight out of 29 patients had either a driveline infection or a device‐specific infection. After device decommissioning, 88% of these patients developed driveline exit site or minithoracotomy infection at a median of 840 days after device decommissioning.

As discussed above, 50% of the patients in the RESTAGE‐HF (Remission from Stage D HF) study,^32^ a multicenter cohort of nonischemic dilated cardiomyopathy (NICM) patients who received an aggressive prospective protocol coupled with mechanical unloading with neurohormonal therapy, had successful explantation of LVAD. The long‐term survival free of HF in these patients was 90% at 1 year and 77% at 2 years postexplant. This study, which included predominantly patients who had their entire LVAD explanted, parallels the analysis by Gerhard et al.^31^ These results suggest that device decommissioning is not associated with higher rates of recurrent HF.

In a recent systematic review of 44 studies (85 patients) examining patient outcomes of device withdrawal by minimally invasive pump decommissioning as compared to reoperation for pump explantation, there were similar rates of patient survival, driveline infection, and cerebrovascular accidents at a median follow‐up of 1 year.^33^ Therefore, device decommissioning may be a feasible alternative to device removal in patients on LVAD therapy with recovery of heart function.^34^, ^35^, ^36^ However, there is no current consensus regarding the optimal anticoagulation strategy in this unique patient population as there is little known about optimal long‐term thromboembolic risk in these patients and no long‐term data is available.

In another evaluation of 53 patients, weaned patients with end‐stage NICM post‐LVAD wean as the underlying cause for VAD implantation revealed 5‐ and 10‐year postexplant survival probabilities (including after heart transplant [HTx] survival for those with HF recurrence) of 72.8 ± 6.6% and 67.0 ± 7.2%, respectively.^23^ Assessment of postweaning survival only from HF recurrence or weaning‐related complications revealed higher probabilities for 5‐ and 10‐year survival, reaching 87.8 ± 5.3% and 82.6 ± 7.3%, respectively. Thus, patients weaned from LVADs appeared not to be at a higher risk for death in comparison with those who underwent HTx, even if the underlying cause for LVAD implantation was chronic cardiomyopathy and not one of the more often reversible cardiac diseases such as acute myocarditis, postcardiotomy HF, or peripartum cardiomyopathy.

Thus, the survival probability of our LVAD‐explanted patients with NICM as the underlying cause for LVAD implantation was better than that of patients with the same underlying cardiac disease who could not be weaned from their LVAD. Postexplant HF recurrence appears to be related to the duration of HF before LVAD implantation, and a preimplant history of HF > 5 years can be a relevant risk factor for postweaning HF recurrence.^37^, ^38^ The influence of the etiology of the underlying cardiac disease responsible for HF development before LVAD implantation on postweaning patient outcomes is barely known. However, with the option of HTx for patients with postexplant HF recurrence, the 5‐year survival probability of our weaned patients with idiopathic dilated cardiomyopathy as the underlying cause for LVAD implantation, a disease that for a long time was considered to be almost irreversible, reached nearly 80%, suggesting that LVAD explantation should be considered in all patients with relevant cardiac recovery, not only in those with potentially more reversible cardiac diseases.

In a study by Dandel et al.,^39^ at the time of evaluation, 33 (70.2%) of the 47 patients with end‐stage CM before VAD implantation, who were weaned since March 1995 from their VADs after different degrees of cardiac recovery, were alive. Kaplan–Meier estimates of overall survival after VAD removal (including post‐HTx survival for those with HF recurrence) revealed probabilities of 71.4 ± 7.1% and 65.7 ± 7.6% for 5‐ and 10‐year survival, respectively. Of 14 patients who have died to date, nine (64.3%) died due to different causes not related to VAD removal. The vast majority of patients with HF recurrence underwent HTx and thus assessment of postweaning survival from HF recurrence or weaning‐related complications by Kaplan–Meier estimates revealed high probabilities for 5‐ and 10‐year survival, reaching 88.4 ± 5.7% and 85.8 ± 6.0%, respectively. There was no difference between groups in the Kaplan–Meier estimates of overall survival for patients with and without HF recurrence after VAD removal (10‐year survival 64.7 ± 11.6% and 67.1 ± 9.7%, respectively; *p* = .76). Kaplan–Meier estimates of transplant‐free survival after VAD removal revealed probabilities of 68.9 ± 7.6% and 61.6 ± 8.6% for 5‐ and 10‐year survival, respectively. The complications related to VAD removal were uncommon. Infection was a major problem in five patients, but in four of these, it was already present before VAD removal.

To date, postweaning recurrence of HF occurred in 17 (36.2%) of the evaluated patients suffering from CM before VAD implantation. Myocardial dysfunction with progressive LV dilation but not the new appearance or aggravation of pre‐existing mitral regurgitation (MR) appeared to be the cause for the recurrence of HF symptoms. Kaplan–Meier estimates revealed a probability of 66.0 ± 8.2% for 5‐year freedom from HF recurrence after VAD removal. In the patient group that underwent VAD removal before March 2002, HF recurred in 14 (45.2%) of 31 patients. Of these HF recurrences, nine (64.3%) occurred during the first year after weaning. In the group weaned thereafter, only three (18.8%) of the 16 patients showed HF recurrences, which all arose late, beyond the fourth postweaning year. There were no significant differences in postweaning cardiac stability between patients who were weaned from continuous flow LVADs and those weaned from pulsatile LVADs. Of the 17 patients with HF recurrence, 15 underwent HTx, one patient (65 years of age at HF recurrence) received another LVAD designed as a chronic mechanical circulatory support, and two patients died suddenly at 2.1 and 5.7 years after LVAD removal, respectively.

## 4 LVAD PATIENT RECOVERY CHARACTERISTICS

According to INTERMACS, 26% of patients with LVADs were listed for cardiac transplantation (bridge to transplant—BTT), 43% as destination therapy (DT), and 30% as bridge to decision. However, less than 1% of patients had sufficient recovery of LV systolic function to lead to LVAD explantation.^18^ There was a similar explanation rate reported in the Momentum 3 trial 5‐year outcomes study of 0.8% at 5 years.^3^ In centers that actively screen for myocardial recovery, defined as LVEF > 40% and LV end‐diastolic diameter (LVEDD) < 6 cm, successful explantation has been reported in as many as ~18%–20% of patients.^40^

Independent predictors of recovery include: age <50 years, etiology of nonischemic cardiomyopathy, and time from cardiac diagnosis <2 years, absence of implantable cardiac defibrillators, creatinine ≤1.2 mg/dL, and LVEDD < 6.5 cm (*p* < .0001). In a cohort of 190 patients with LVADs, a weighted score was developed (I‐CARS score) to effectively stratify patients based on their probability of recovery. INTERMACS Cardiac Recovery Score (I‐CARS) was validated and exhibited good performance (area under the curve [AUC]: 0.94; 95% CI: 0.91–0.98). Those who met the I‐CARS criteria were reported in INTERMACS to have an 86% 1‐year survival after LVAD explant. A total of 21 patients (11% of the total cohort) achieved myocardial recovery.^6^

## 5 PROTOCOL FOR LVAD EXPLANTATION AND ASSESSMENT OF MYOCARDIAL RECOVERY

It is important to note that assessment of recovery is not uniformly or rigorously performed. Currently, there is no consensus or protocol to either assess recovery or wean LVAD support for those considered to be candidates. Several centers have published their experiences, with the most studied approaches described by Birks et al.^30^, ^41^ and Dandel et al.^23^, ^42^ Their protocols are primarily based on performing serial echocardiograms (ECHOs) to assess LV unloading and return of cardiac function. Patients who were considered and evaluated for explant were all on good doses of GDMT and did not require inotrope use. Frazier et al.^22^ championed a weaning strategy based on the cardiac cycle. When adequately unloaded (i.e., normal LV dimensions and minimal MR), patients are serially evaluated at minimal pump speeds for normalization of aortic valve opening time. With this reconditioning approach, they were able to successfully remove pumps from 27 patients.

**Figure 1:**
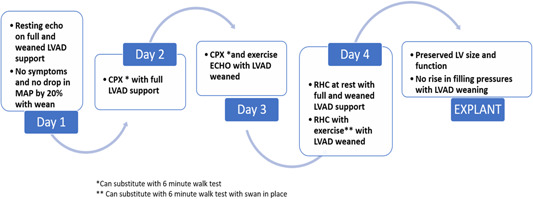
Proposed protocol for weaning and assessment of recovery. CPX, cardiopulmonary exercise test; ECHO, echocardiogram; LV, left ventricle; LVAD, left ventricular assist device; MAP, mean arterial pressure; RHC, right heart catheterization.

A three‐step approach to assess cardiac reserve in patients with HMII LVADs has been described (Figure 1). This included echocardiography, cardiopulmonary exercise testing, and right heart catheterization.^43^ More specifically, each of these tests was performed at rest and peak exercise, with patients fully supported by their LVADs. This was followed with weaning over 4–7 days, with a reduction of speed of HMII to only 6200 rpm to prevent retrograde flow through the outflow graft. The weaning protocol was stopped if the patient became symptomatic or the mean arterial pressure dropped by 20%. In this study, six patients completed the protocol. Fifty percent of patients (3/6) were selected for explant after demonstrating preserved LV size and function, and there was no rise in filling pressures with LVAD weaning. Importantly, peak *V*O_2_ and peak CO_2_ remained diminished in all patients and could not be utilized in this selection process. Right ventricular function improved in all six patients and was normal in the three explanted patients. HF medications were continued after explant and all three patients remained asymptomatic with normal LV function at 14–32 months of follow‐up. The caveat to this protocol is that not all centers are able to perform the outlined studies; therefore, generalizability is more difficult. It is reasonable to consider 6‐min walk testing as a substitute for *V*O_2_ Max testing and walking patients with swans in place in the critical care unit instead of an exercise right heart catheterization in centers that are not equipped to perform the standardized exercise hemodynamics.

Long‐term follow‐up in patients from RESTAGE HF (a multicenter prospective nonrandomized study) was recently published. It involved a standardized approach to both the evaluation for recovery and the weaning of LVAD support. Forty patients with HMII LVADs were enrolled from six centers. All with nonischemic CM and LVEF < 25% preimplant and HF duration <5 years.^32^ Postoperatively, LVAD speed was optimized with echocardiographic guidance to achieve LVEDD < 6 cm and to reduce the degree of MR. GDMT was implemented with the aim for it to be initiated immediately after the weaning of inotropic support after LVAD implantation once there was adequate end‐organ recovery. Medications were titrated to a mean arterial pressure >60 mm Hg if the patient remained asymptomatic with adequate renal function. Targeted doses of GDMT included lisinopril 20 mg twice daily or losartan 150 mg daily, carvedilol 50 mg twice daily, spironolactone 25 mg daily, and digoxin 0.125 mg daily. ECHOs were performed at Week 6 and at Month 4, 6, and 9 visits, combined with a 6‐min walk test. At the Month 12–18 visit, the low‐speed ECHO was done depending on the improvement in cardiac function seen. A full ECHO was performed at the patient's baseline speed, and then, with an international normalized ratio (INR) > 2, the pump speed was reduced to 6000 rpm in increments of 1000 rpm over 1–2 min. A limited ECHO was performed for 5 min and then a full ECHO was performed for 15 min at 6000 rpm. If the subject tolerated the speed of 6000 rpm for 15 min, a 6‐min walk was performed at 6000 rpm, followed by a repeat ECHO. Before explant, a cardiopulmonary exercise test was performed at 6000 rpm, and right ± left heart catheterization was performed at baseline speed and at 6000 rpm for 15 min.

Explant patients had to meet the following criteria measured at speeds of 6000 rpm for 15 min:

1. LVEDD < 60 mm, LV end‐systolic dimension < 50 mm, LVEF > 45%.
2. LV end‐diastolic pressure or pulmonary capillary wedge pressure ≤15 mm Hg.
3. Resting cardiac index > 2.4 L/min/m^2^.
4. Optional—Maximal oxygen consumption with exercise >16 mL/kg/min.

Approximately 50% of the patients enrolled in RESTAGE HF were able to be weaned successfully and survival free from LVAD or transplantation in those explanted was 90% at 1 year and 77% at 2 and 3 years.

**Figure 2:**
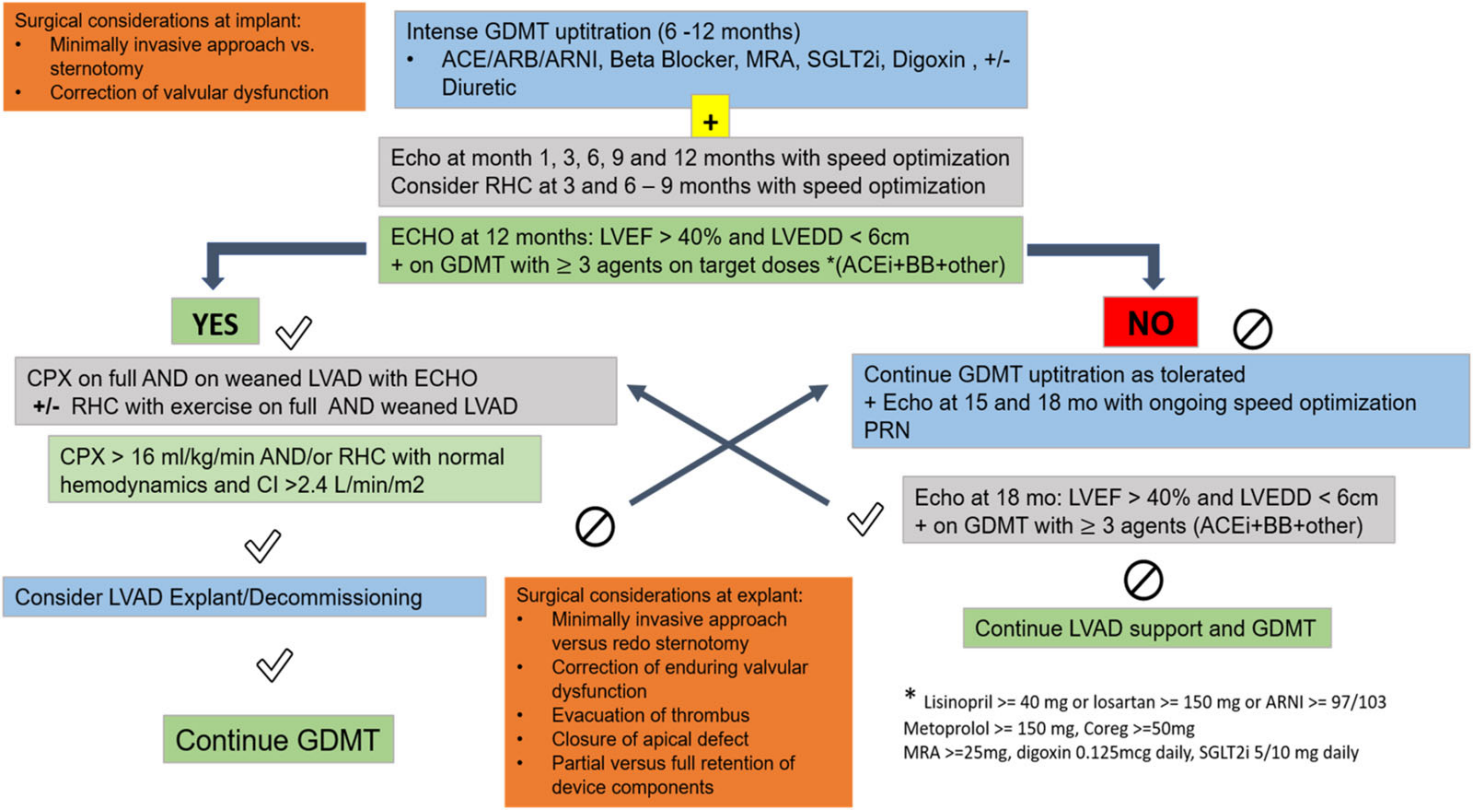
Proposed algorithm for medical optimization, recovery assessment, and surgical considerations. ACEi, angiotensin‐converting enzyme inhibitors; ARB, angiotensin II receptor blocker; ARNI, angiotensin receptor‐neprilysin inhibitor; BB, β‐blocker; CI, cardiac index; CPX, cardiopulmonary exercise test; ECHO, echocardiogram; GDMT, guideline‐directed medical therapy; LVAD, left ventricular assist device; LVEDD, left ventricular end‐diastolic diameter; LVEF, left ventricular ejection fraction; RHC, right heart catheterization; SGLT2i, sodium‐glucose cotransporter 2 inhibitor.

It is important to note that current device and medical therapy has evolved and we do not have enough data with HM3 devices and patients on GDMT that includes Entresto and Jardiance regarding myocardial recovery/remission. HM3 devices have a much more favorable hemocompatibility profile and there may be higher tolerance to speed lowering as far as a thromboembolic event rate in these patients. A randomized clinical trial—ARIES—is underway to compare the vitamin K antagonist regimen with aspirin versus vitamin K antagonist with placebo. The ARIES HM3 pump IDE study (https://classic.clinicaltrials.gov/ct2/show/NCT04069156). In Figure 2, we propose a protocol for optimizing and assessing myocardial recovery in LVAD patients considering described data in this field.

## 6 EXPLANT METHODS AND IMPLICATIONS

When LVADs are to be utilized as a bridge to recovery, there are surgical considerations that begin at the time of original device implantation. Concomitant valvular interventions at the time of device implant have been described and promoted for the traditional BTT or DT settings.^44^, ^45^, ^46^ Optimizing the function of the recovered heart at the time of explant would also necessitate correction of valvular heart dysfunction at the time of device implant.^47^ This allows the LVAD to work at its most optimal and promotes reverse remodeling during therapy.

Aortic insufficiency (AI) must be corrected for suitable function of the LVAD.^48^, ^49^ Correction of AI includes valve replacement and valve repair that may range from annuloplasty and leaflet reconstruction to suture coaptation of the leaflets and completely oversewing the valve.^50^ Without sufficient data to promote a particular approach, it may be theorized that replacement with a bioprosthetic valve may be best for those patients to be bridged to recovery who have AI, aortic stenosis, or a mechanical valve prosthesis.^47^

The correction of tricuspid regurgitation (TR) remains controversial. While regaining competency of the tricuspid valve at the time of device implant leads to shorter durations of inotropic support and hospital length of stay, there is no improvement in outcome as compared to LVAD implant alone in the BTT and DT populations.^51^ When recovery and explant are the goal, it stands to reason that repair of the TR would support right ventricular reverse remodeling and function, thereby improving the overall function of the recovered heart.

In HF, due to remodeling of the LV caused by annular dilatation, papillary muscle distortion, and chordal tethering, MR is commonly encountered.^52^ While the LVAD provides unloading of the LV, reverse remodeling of the LV is not guaranteed with therapy. Mitral valve intervention with repair or replacement decreases pulmonary vascular resistance safeguarding the right ventricle, but these surgical interventions add time and complexity to the LVAD implant procedure. A transapical edge‐to‐edge repair of severe MR would overcome time and complexity confounders but would not be appropriate for surgical correction of severe annular dilation, likely requiring a left atrial surgical approach.^53^, ^54^

The extent of surgical intervention at the time of LVAD explant after recovery must also be considered. Cardiopulmonary bypass without cardiac arrest is utilized to support the heart at the time of explanation. Surgical planning and approach for successful LVAD explant must also include addressing any thrombus that may be present in the left ventricle as well as enduring structural heart issues.^47^

The surgical team must decide to approach the explant of the LVAD through a redosternotomy or through minimally invasive approaches. A redosternotomy is associated with increased blood transfusion requirements and injury to the heart that may threaten the success of LVAD explant of a recovered heart, so adaptation of minimally invasive techniques of LVAD explant has occurred. Minimally invasive approaches to repair the left ventricular apex will commonly utilize femoral and outflow graft arterial access and femoral venous access for cardiopulmonary bypass without cardiac arrest.^47^

When explanting the LVAD, closure of the defect in the LV must occur utilizing an apical plug, a patch, or primary closure. The defect in the LV apex for the inflow may be repaired primarily after explant by approximating the muscular edges of the LV with a series of interrupted sutures or with a running suture line. When a flexible sewing ring has been utilized at the time of LVAD implant, the edges of the sewing ring may provide a buttress for the suture line. For less flexible sewing rings, the sewing ring may be removed and the defect in the LV apex for the inflow may be repaired primarily or the sewing ring may be left in place and the defect in the LV apex for the inflow may be closed using a plug fashioned from surgical felt with or without a biologic covering or cap. The plug is positioned within the sewing ring and secured eliminating the need for an extensive LV repair. A more rigid sewing ring left in place and closed with a plug will provide access if the LVAD needs to be replaced.^47^, ^55^ After closure of the left apical defect the outflow graft may be transected at its insertion point on the ascending aorta either by utilizing a surgical stapler or by oversewing with suture.

When embracing minimally invasive surgical techniques, complete removal of the device and outflow graft is far more complex and potentially not possible. Therefore, retention of portions or of the entire pump with inflow and outflow grafts may be necessary. To avoid a midline sternotomy, multiple smaller incisions overlying key components of the LVAD may be made to access the LVAD, inflow, and outflow cannula. When the outflow graft has been used for arterial access for cardiopulmonary bypass, the graft may be oversewn or stapled at the cannulation site leaving a small, retained portion of the outflow graft. The most minimally invasive technique described avoids cardiopulmonary bypass and involves leaving the entire LVAD intracorporeally and cutting the driveline. This results in a contained pump thrombus. When complete removal of the device and outflow graft is not accomplished, rare complications such as infection of the residual graft material or thromboembolic incidents have the potential to occur.^56^, ^57^, ^58^

## 7 MEDICAL MANAGEMENT AND MONITORING AFTER LVAD EXPLANTATION OR DECOMMISSIONING

### 7.1 HF GDMT

The pioneering work of Birks et al.^5^, ^41^ showed that device explantation can be achieved in 73% of selected patients with the combination of maximal GDMT and clenbuterol. Thirty‐six percent of patients who had LVAD explantation had sustainable remission from HF recurrence.^32^ To date, a consensus among the MCS community on how to monitor patients post‐LVAD explantation or decommissioning has not been developed. However, there are several monitoring strategies that are reasonable to utilize. First, echocardiography can be used as the primary tool for monitoring cardiac function after LVAD removal. Various protocols are possible and could include initially having daily ECHOs in the first week after weaning, following to weekly, and then a change in frequency once discharged from the hospital from monthly to every 6 months. Other testing could include an assessment of exercise tolerance, evaluated by a maximum (symptom‐limited) incremental treadmill exercise test or cardiopulmonary test to define exercise tolerance and capacity objectively. However, this has not been recommended before 2–3 months after explantation or decommissioning. Lastly, routine blood work including NT‐proBNP of BNP plasma levels can also be measured.^39^

## 8 ANTICOAGULATION AND ANTIPLATELET THERAPY

There are currently no consensus recommendations for antiplatelet or anticoagulation for patients who undergo LVAD explantation or decommissioning following either myocardial recovery or a device‐related complication. In a recent scoping review, 15 studies were identified that outlined anticoagulation strategies with regimens consisting of combinations of vitamin K antagonists, and unfractionated heparin and/or bivalirudin.^59^ There were four studies that maintained that patients should continue anticoagulation for 3 months in patients who received device explantation. If the apical ring and plug were retained postexplantation, the use of vitamin antagonists for 3 months and aspirin indefinitely was recommended by two studies. In patients who underwent device decommissioning, four groups reported stopping anticoagulation after the procedure, while five groups continued anticoagulation with vitamin K antagonist for at least 4 months postdevice decommissioning. Five groups reported using lower INR targets following LVAD decommissioning. While the presence of retained device material or the use of plugs may be a risk factor for thromboembolic complications, none of the patients in the case reports or series included experienced a thromboembolic complication. However, Baldwin et al.^60^ reported four patients who suffered from ischemic stroke with two of the events directly related to either the hemostatic clip or manipulation of the outflow graft. The risk of future thromboembolic events is likely to be related to the amount of retained foreign materials following device explantation or decommissioning.

Other complications have also been reported including infection and recurrent HF. Sepsis and driveline infections were reported and in one case leading to complete LVAD explantation. These are similar findings to the European Registry for Patients with Mechanical Circulatory Support (EUROMACS) study, which included 45 patients, with a median follow‐up period of 26 months following LVAD explantation and reported one patient death due to sepsis. As long‐term results postexplantation or decommissioning become available, reliable parameters to predict outcomes can be identified.^61^

## 9 FUTURE DIRECTION AND CONCLUSION

The HF pandemic continues to grow as does the data showcasing the benefits of ventricular assist devices. The significant improvement in morbidity and mortality provoked the continued technological development of these devices aimed at improving the adverse event risk profile. As technology continues to evolve the focus on these devices as catalysts to recovery along with GDMT and other structural and electrophysiological interventions is of the essence. This entails both early assessment of potential for recovery and early utilization before end‐organ dysfunction, and further remodeling to myocytes occurs. Changing the VAD communities' approach to VAD support will lead to further recovery and remission. In summary, LVAD therapy combined with GDMT can facilitate remission and in some cases lead to partial or full myocardial recovery. In this review, we have included a pathway for cardiac‐assisted remodeling utilizing GDMT, unloading and reloading. Furthermore, we defined criteria and methodologies for explant and maintenance of remission.

## CONFLICT OF INTEREST STATEMENT

The authors declare no conflict of interest.

## Data Availability

Data sharing is not applicable to this article as no data sets were generated or analyzed during the current study. There are no outside sources for any data presented in this manuscript.
